# The GRID Technique: Current Status and New Trends

**DOI:** 10.6028/jres.105.004

**Published:** 2000-02-01

**Authors:** Michael Jentschel, Hans G. Börner, H. Lehmann, C. Doll

**Affiliations:** Institute Laue-Langevin, F-38042 Grenoble, France; Technische Universität München, D-85748 Garching, Germany

**Keywords:** atomic collisions, gamma ray spectroscopy, interatomic potentials, nuclear state lifetimes, nuclear structure

## Abstract

In the GRID technique one measures Doppler-broadened line profiles of γ transitions using the high resolution crystal spectrometers GAMS, which are installed at the high flux reactor of the ILL Grenoble. One of the essential applications of this technique is the measurement of nuclear state lifetimes. In the present contribution the precision and the principal limits of the technique are discussed.

## 1. Introduction

**Fig. 1:**
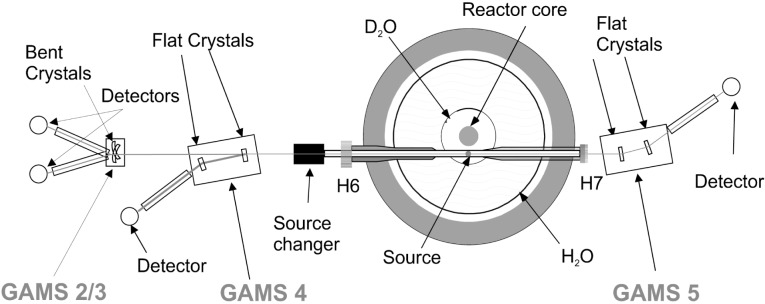
Experimental set-up of the crystal spectrometers installed at the in-pile tangential beam tube of the high flux reactor of the ILL.

During the last three decades, crystal spectrometers have been developed and installed at the high flux reactor of the Institut Laue-Langevin. Currently, three devices are available: the single bent crystal spectrometer GAMS2/3 and the two double flat crystal spectrometers GAMS4 and GAMS5. They are installed in line to an in-pile tangential beam tube. In this beam tube targets can be placed at a distance of 55 cm to the reactor core where the thermal neutron flux equals 5×10^14^ s^−1^ cm^−2^. A general scheme of the experimental set-up is shown in Fig. 1. The combination of the intense γ flux and the ultra high resolution of the double flat crystal spectrometers GAMS4 and GAMS5 has allowed the development of the Gamma Ray Induced Doppler broadening technique. Currently, this technique is widely used for nuclear state lifetime measurements and the study of atomic collision processes in solids [2]. It is the purpose of this contribution to give a brief description of the technique itself. Furthermore, an attempt to estimate quantitively the sensitivity of the technique in nuclear state lifetime measurements is presented.

## 2. GRID Basics

A detailed description of the GRID technique has been given in a series of publications [1,2,3] and therefore only the basic ideas should be presented here.

Thermal neutron capture produces an excited nucleus, which deexcites either by γ emission or other decay processes. Whenever an emission process takes place, the nucleus is recoiling due to momentum conservation. In the particular case of a γ cascade this means that the measured energy of any secondary γ ray might be Doppler shifted by a recoil induced during a prior emission of another γ ray. Due to the spatial distribution of the recoil directions, it is a Doppler broadening, which will be obtained. The broadening depends on the velocities of the recoiling nuclei at the time of the secondary emission and therefore on three factors: i) the initial recoil velocity, ii) the time between the initial recoil and the emission of the observed γ ray e.g., the lifetime of the intermediate level and iii) the slowing down of the recoiling particles. The measurement of the Doppler-broadening allows extraction of information on one of these quantities, assuming the other two to be known.

For illustration, we consider first the very simple case of a two step γ cascade, where the intermediate level is fed to 100 % by a primary transition of energy *E*_γ1_. As the initial recoil velocity is known to be υ_R_ = *E*_γ1_/*Mc*, where *M* is the mass of the recoiling isotope and *c* the velocity of light, the Doppler-broadened lineshape of the secondary transition *E*_γ2_ depends only on the lifetime of the intermediate nuclear state and the slowing down of the recoiling atoms.

In order to have a description for the slowing down process of the recoiling atoms Jolie et al. [2, 4] have assumed a model, in which the slowing down process is assumed to be isotropic. Here, the description of the recoil motion is reduced to the determination of the time dependence of the average velocity υ(*t*) of all recoiling atoms. This assumption is strongly based on the use of powder targets. In this case, the γ rays are emitted from a large number of microcrystals and any microscopic anisotropy of the slowing down process is averaged out due to the random alignment of the microcrystals. Assuming υ(*t*) to be known, the Doppler-broadened γ lineshape is given by the equation

$$\begin{array}{c} I_{D}\left(E\right)dE=C\int_{0}^{\infty }\exp\left(-t/\tau \right)dt\\ \times \;[\arctan\;\frac{\Gamma }{2}\left\{E-E_{\gamma 2}\left(1-\frac{\upsilon \left(t\right)}{c}\right)\right\}-\arctan\;\frac{\Gamma }{2}\left\{E-E_{\gamma 2}\left(1+\frac{\upsilon \left(t\right)}{c}\right)\right\}], \end{array}$$ (1)

where *Γ* represents the natural width and *τ* the lifetime of the depopulated nuclear state. The calculation of υ(*t*) was first solved within the so-called Mean Free Path Approach (MFPA) [4]. Later also other approaches like Molecular Dynamics simulations [5] or Restricted Molecular Dynamics [8] have been applied.

If there are no other sources of additional broadening, Eq. (1) and a known function υ(*t*) would allow extraction of a nuclear state lifetime and a direct comparison with the experiment. However, there exist two further factors which must be taken into account: i) the instrumental response function and ii) the Doppler-broadening due to thermal motion. Both of them can be determined experimentally. In fact, with the double flat crystal spectrometers GAMS4 and GAMS5 the determination of the instrument response function is possible with very high accuracy in the so-called nondispersive mode (for more details see Ref. [2]. The thermal Doppler-broadening can be determined by scanning a transition, which depopulates a very long lived state (τ > 10 ps). In this case, it can be assured that the state is mostly depopulated, when any recoil motion is slowed down and the atom moves with typical thermal velocities. Comparing such a scan with a nondispersive scan of the same transition (yielding the instrument response function) a direct extraction of the thermal Doppler-broadening is possible. The knowledge of the thermal Doppler broadening allows extraction of a target temperature *T*, which will enter into the calculation of the initial and final values of the function υ(*t*) within the different slowing down models. Consequently, the Doppler-broadened lineshape *I*_D_(*E*) will depend on the target temperature. The total lineshape, which needs to be compared to an experiment can be written as

$$I_{T}(E)=I_{D}(E)*R(E),$$ (2)

where *I*_T_(*E*) denotes the total lineshape obtained as a convolution of a Doppler-broadened lineshape *I*_D_(*E*) and the instrumental response function *R*(*E*). Assuming the knowledge of υ(*t*) it has been shown that it is possible to extract lifetimes of nuclear states in the range of 10^−15^ s to 10^−11^ s using the least squares fitting routine GRIDDLE [9]. Also the inverse problem can be considered if the lifetime *τ* is known and the function υ(*t*) has to be extracted, which allows the study of the atomic motion in solids.

## 3. Accuracy

The structure of Eq. (1) suggests that the precision with which a nuclear lifetime is extracted depends on the accuracy of υ(*t*). Due to the low recoil velocities, the theoretical description of the atomic motion can be restricted to classical mechanics, i.e., to the solution of Newton’s equations of motion for point mass particles interacting via a classical interatomic potential.

Within the MFPA the slowing down process is considered to be on average a sequence of binary atomic collisions, where the recoiling atom looses in every collision half of its kinetic energy. Between collisions the atoms are on average assumed to move with constant velocity. The result of this assumption can be summarized as a step function

$$\upsilon_{k+1}\left(t_{k+1}\right)=\frac{1}{\sqrt{2}}\upsilon_{k}\left(t_{k}\right),t_{k}=\sum_{k=1}^{k}\frac{\tau_{k}}{\upsilon_{k-1}},$$ (3)

where τ*_k_* describes the mean free paths between two collisions. They can be calculated knowing the density of the target material and assuming an atomic interaction potential [2].

A more precise approach for the calculation has been introduced to the GRID technique by Kuronen et al. [5], who used Molecular Dynamics (MD) simulations for the calculation of υ(*t*). This technique is quite common in the computer simulation of ion beam solid interaction [11] and is exact within classical mechanics. The motion of the recoiling atom is considered within a simulation cell containing *N* atoms, where *N* = 500 to 1000. Newton’s equations of motion of all *N* atoms, interacting via an interatomic potential, are simultaneously integrated. However, the computational procedure is rather complicated as *N*(*N* – 1)/2 equations have to be solved. Therefore, a slightly simplified approach (Restricted Molecular Simulations) has been introduced to the GRID-technique [8]. It can be argued that the interaction of atoms far away from the recoiling nucleus is negligible. This allows restriction of the integration to only those equations which describe the interaction of atoms within a certain sphere surrounding the recoiling atom.

In both MD and RMD simulations, a large number of recoil events (typically several thousands) is calculated and an average velocity υ(*t*) deduced afterwards. As these two models do not directly calculate the average velocity they can be used to test the model of an isotropic slowing down. This can be done by replacing Eq. (1) by the following expression

$$\begin{array}{c} I_{D}(E)=C_{1}\sum_{j=1}^{M}\int_{0}^{\pi }d\theta \;\sin\;\theta \\ \times \int_{0}^{\infty }dt\;\frac{\exp\left(-t/\tau \right)}{{\left[E-E_{\gamma 2}\left\{1+c^{-1}\upsilon_{j}\left(t\right)\cos\;\theta \right\}\right]}^{2}+{\left(\Gamma /2\right)}^{2}}. \end{array}$$ (4)

Here *C*_1_ is a normalisation constant, υ*_j_*(*t*) the velocity of the *j*-th recoiling atom and *θ* is the angle under which the *j*-th particle moves with respect to the direction of observation. The summation goes over *M* recoil events. The first integration takes account for the isotropic alignment of the microcrystals and the second integration introduces the emission probability of the second γ ray. Comparing lineshapes, which were calculated via Eq. (4) to lineshapes calculated via Eq. (1) has shown that the isotropic model can be used for polycrystalline targets.

**Fig. 2:**
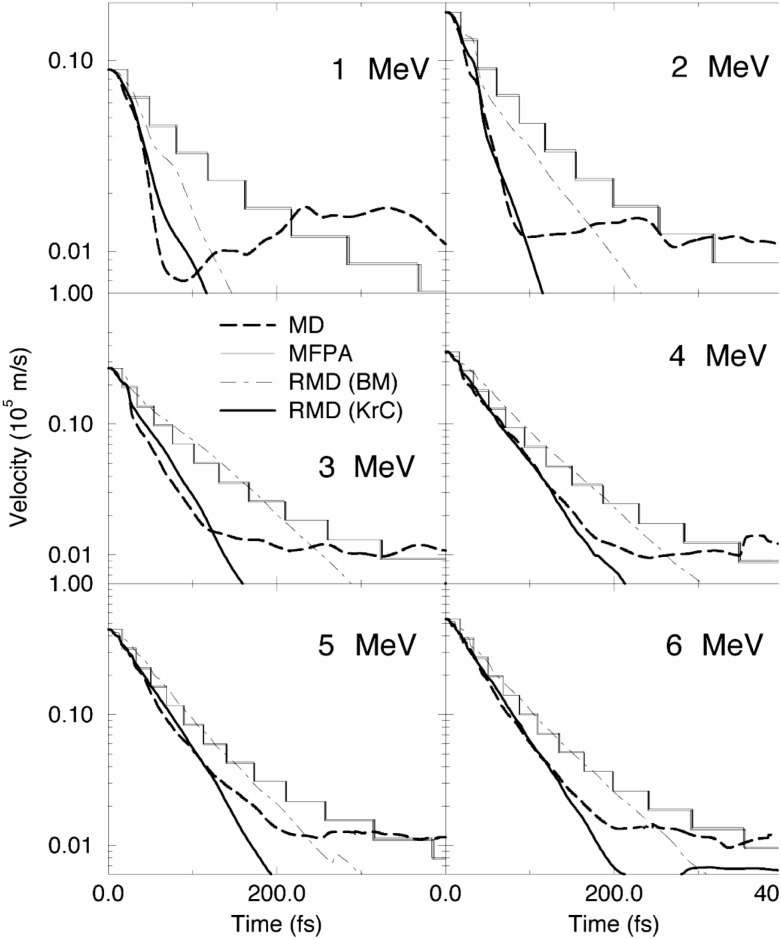
Time dependence of the average velocity of recoiling ^36^Cl-atoms in a NaCl target calculated with three different slowing down models. The energies in every plot correspond to the energy of the γ ray, which initiated the recoil. The thick dashed lines shows the result from Molecular Dynamics simulations, the full line the Mean Free Path Approach, and the dash-dotted line the Restricted Molecular Dynamics simulations. In all three of them the Born-Mayer potential has been used to model the repulsive atomic interaction. The thick full line describes the results obtained from Restricted Molecular Dynamics simulations using a screened Coulomb potential (KrC).

In all three models mentioned above, the basic input is defined by the assumption of an interatomic potential. Essentially two potentials are used: The MFPA is based on a Born-Mayer type repulsive potential [7]. The MD and RMD simulations allow implementation of any type of potential, but mainly screened Coulomb type potentials are used [11]. There exists only a sparse knowledge on the atomic interaction in solids at kinetic energies of several hundreds of eV. In Fig. 2, the function υ(*t*) was calculated for ^36^Cl atoms recoiling in NaCl, using different theoretical approaches. Within a time interval of up to 2×10^−13^ s the choice of the interatomic potential seems to introduce the dominating uncertainty in the slowing down description. Later, the difference of the slowing down models becomes dominating.

In order to quantify the influence of the uncertainty in the slowing down description on the lifetime determination up to 5×10^−14^ s, the function υ(*t*) was approximated by an exponential behavior υ(*t*) = υ_0_ exp(–*t*/*T*). The parameters υ_0_ and *T* represent the initial recoil velocity and the average slowing down time, respectively. Although this model is rather simple, it gives a surprisingly good fit to the simulated υ(*t*) curves for time intervals up to 2×10^−13^ s. Using this approximation the function *I*_D_(*E*) can be analytically calculated to be

$$\begin{array}{c} I(E)dE\\ =\frac{C}{T/\tau -1}{\left(\frac{1}{\upsilon_{0}}\right)}^{T/\tau -1}\left[\upsilon_{0}^{T/\tau -1}-{\left|c\left(1-\frac{E}{E_{\gamma 2}}\right)\right|}^{T/\tau -1}\right]dE, \end{array}$$ (5)

where *C* denotes a normalization constant. The nuclear state lifetime enters into equation [5] only as τ/*T*. From this it can be concluded that any relative uncertainty of the average slowing down time *T* gives the same relative uncertainty for the lifetime extraction. An analysis of the calculations presented in Fig. 2 yields an average variance of *T* and therefore *τ* of about 36 %. A similar result was obtained in [2], where a detailed comparison of several simulations has been performed. According to the calculations presented in Fig. 2 it seems that the validity of the interatomic potential is the dominating uncertainty. However, all comparisons so far have been performed for lifetimes up to 10^−13^ s and a check for longer lifetimes is still missing. This is mainly caused by the bad numerical efficiency of MD simulations.

Up to now considerations have been based on the assumption of a fully primarily fed nuclear level of interest. In the more general (and more frequent) case, the level of interest is fed by several γ cascades passing several intermediate levels. Here, it is difficult to estimate the velocity distribution of the recoiling nucleus, when the level of interest is reached. If the intensities of all γ transitions and the lifetimes of all intermediate levels are known, this distribution can be calculated. In most of the cases this does not apply and the uncertainty of this missing knowledge has to be quantified with respect to the extraction of nuclear state lifetimes from Doppler-broadened lineshapes. For this purpose several approaches are used:

A rather conservative estimation can be done within the so-called extreme feeding assumptions. They are based on the comparison of the summary intensities populating and depopulating the level of interest. If the summary intensity of all depopulating transitions is greater then the populating one, the missing populating intensity can be used to formulate the extreme feeding assumptions. In one extreme all missing intensity is assigned to a primary feeding and in the other one it may be assigned to a transition depopulating a very long lived state, which has an energy of about 1 MeV higher then the level of interest. In the first case the velocity distribution contains a maximum possible number of high energetic recoils. This means that in order to obtain a certain given Doppler broadening, the lifetime of the level of interest has to be rather long. In such a way an upper limit for the lifetime estimation can be obtained. In the second case, the velocity distribution contains a maximum number of low energetic recoils, yielding such the lower limit for the lifetime.

**Fig. 3:**
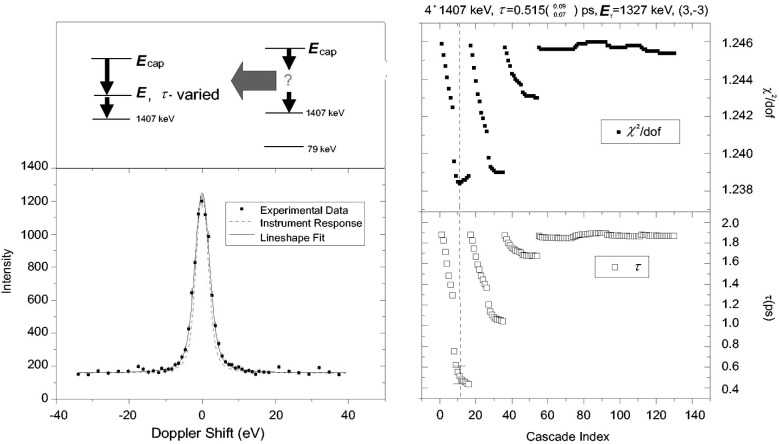
The conception of a two step cascade analysis is demonstrated for a lifetime measurement in ^158^Gd. The unknown feeding of the 1407 keV state is replaced by a two step cascade, where the energy and the lifetime of the intermediate state are varied. For every realisation of this cascade a lineshape fit has been performed and the lifetime and the *χ*^2^ are plotted. A most likely feeding scenario could be identified by the minimum *χ*^2^ value.

Another possibility for the assignment of the missing intensity is given by a so-called *χ*^2^ analysis of the experimental data. Here, a certain parametrization of the missing part of the recoil velocity distribution is chosen. The parameter values are then included in the fit procedure for the evaluation of the experimental data. A typical parametrization is given by a two step cascade (see Fig. 3). Here, all missing populating intensity is assigned to a two step-cascade above the level of interest and the lifetime and the energy of the intermediate level are used as parameters. For illustration the two step cascade parametrization has been applied to the evaluation of data taken with the 1327 keV transition in ^158^Gd (Fig. 3). As the evaluation is based on the increase of parameters within a least square fit, the data have to yield sufficient statistical significance to allow a constructive optimization of the recoil velocity distribution. However, any result obtained within this approach will fall within the limits of the extreme feeding assumptions.

## 4. Sensitivity

The simple model of an exponential slowing down can be extended to include thermal motion. This can be done by folding Eq. (5) additionally with a Doppler-broadened lineshape, as it results from thermal motion at temperature *T* with a Maxwell-Boltzmann distribution. This takes account for the variance of the initial recoil velocity and the final convergence of the slowing down to an average thermal velocity. The result *Ĩ*_D_(*E*, τ) can not be given in analytical form. However, numerically it is easy to handle and can be used to demonstrate the sensitivity of the GRID technique to the different parameters, which have to be used for an evaluation of a measured lineshape. As the evaluation of the data is done by means of a least square fitting, the following function shall be considered

$$\begin{array}{c} \psi^{2}=\sum_{i}{\left|\frac{{\tilde{I}}_{D}(E_{i},\tau +\Delta \tau )\;{\tilde{I}}_{D}(E_{i},\tau )}{\sqrt{{\tilde{I}}_{D}(E_{i},\tau )}}\right|}^{2}\\ ≃{\sum_{i}\left|\frac{\frac{\partial }{\partial \tau }{\tilde{I}}_{D}(E_{i},\tau )}{\sqrt{{\tilde{I}}_{D}(E_{i},\tau )}}\right|}^{2}. \end{array}$$ (6)

**Fig. 4:**
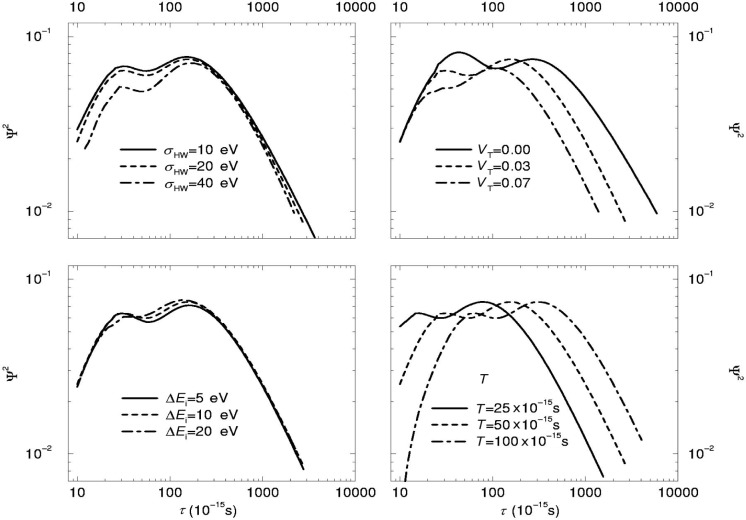
The function *Ψ*^2^ is shown for different parameter sets for the calculation of *Ĩ*_D_(*E_i_*, τ). In every plot one parameter has been varied and compared to a reference calculation (dashed line), which was calculated for *E*_γ_ = 1 MeV, *υ*_R_ = 0.5 × 10^5^ m/s, Δτ/τ = 0.1, *T* = 50 × 10^−15^ s, Δ*E_i_* = 10 eV, σ_HW_ = 20 eV and *υ*_T_ = 0.03 × 10^5^ m/s.

Here, *E_i_* are discrete points with a certain spacing of Δ*E* on the energy axis representing the analog to experimental data sampling within a scan. The function *Ψ*^2^ has the same analytical structure as the partial derivative of the optimization function *χ*^2^ in a least square fit. Thus, an increase in the values of *Ψ*^2^ means an increase in sensitivity in lifetime extraction. In Fig. 4 the dependence of *Ψ*^2^ has been calculated as function of *τ* for assumed parameters of υ_0_ = 0.5 Å/fs for the initial velocity Δτ/τ = 0.1 for a desired precision of the lifetime measurement, *T* = 50 fs for the average slowing down time, Δ*E*_i_ = 10 eV for the scan spacing and *υ_T_* = 0.03 Å/fs for the thermal velocity and *σ*_HW_ = 20 eV as FWHM of the instrument response function. In general it can be outlined that the highest sensitivity of the technique is given in the lifetime region of 10^−14^ to 10^−12^ s. At lower lifetime values the decreasing sensitivity will be compensated by an increasing influence of the natural width of the intermediate level. For lifetimes longer than one picosecond the technique loses strongly in sensitivity. This can partly be compensated by increased statistics. For example, for the extraction of a lifetime of *τ* ≈ 2 × 10^−12^ there have to be taken twice as many scans as for *τ* ≈ 1 × 10^−12^.

Using the function *ψ*^2^ the influence of the instrument resolution, the target temperature and the slowing down time on the sensitivity have been investigated. Following the upper left plot in Fig. 4 the instrument resolution shows no substantial influence within the typical ranges of variation. The thermal velocity *υ_T_* (upper right plot) appears to be a strongly limiting factor for the sensitivity to lifetimes longer than 10^−12^ s, as with increasing target temperature more and more statistics is required to extract a certain lifetime. The scan interval (lower left plot) appears to be of no significance for the sensitivity. A strong influence comes from the slowing down time. As expected from Eq. (2) an increase in the slowing down time would allow to extend the technique towards longer lifetimes.

## 5. Conclusions

The GRID technique has been shown to be a sensitive experimental technique for the investigation of nuclear state lifetimes in the range of 10^−15^ to 10^−12^ s. It requires the knowledge of atomic motion with several hundreds of eV kinetic energy and the feeding scenario of the nuclear level of interest.

The relative uncertainty of the theoretical description of atomic motion can be quantified in certain cases. It results mainly from the unknown atomic interaction. Lifetimes in the range of 10^−15^ to 10^−13^ s measured with the GRID technique have the same relative uncertainty as the slowing down description. Recently, efforts have been made to improve the current status and to study explicitly the atomic interaction using the GRID technique itself. This can be done using single crystal targets (Crystal-GRID), which allows a significant increase in the amount of information obtained. Further details can be found in Refs. [12, 13].

An additional uncertainty for the lifetime extraction is caused by an unknown feeding scenario for the level of interest. It can be quantified using the concept of extreme feeding scenarios, which yields the maximum variation of the measured lifetime value as it can be produced by the unknown feeding. Using the approximation of a two step cascade for the missing feeding one can find a most likely recoil velocity distribution and extract the most likely directly from the experimental data. Alternativelly, the missing feeding can be calculated using statistical models of the nucleus [14].
